# The Role of HCG in Implantation: A Mini-Review of Molecular and Clinical Evidence

**DOI:** 10.3390/ijms18061305

**Published:** 2017-06-19

**Authors:** Antonis Makrigiannakis, Thomas Vrekoussis, Emmanouel Zoumakis, Sophia N. Kalantaridou, Udo Jeschke

**Affiliations:** 1 Department of Obstetrics and Gynecology, Medical School, University of Crete, Heraklion 71003, Greece; 2 Department of Obstetrics and Gynecology, Medical School, University of Ioannina, Ioannina 45110, Greece; vrekoussis@gmail.com; 3 Department of Pediatrics, Medical School, University of Athens, Athens 11527, Greece; zoumakis@bioacademy.gr; 4 Second Department of Obstetrics and Gynecology, Medical School, University of Athens, Athens 11528, Greece; sophiakalantaridou@gmail.com; 5 Departments of Obstetrics and Gynecology, Medical School, Ludwig-Maximilians University of Munich, Campus Innenstadt & Campus Grosshadern, Munich 80337, Germany; udo.jeschke@med.uni-muenchen.de

**Keywords:** human chorionic gonadotropin (HCG), implantation, infertility

## Abstract

Embryo implantation is a complex process involving continuous molecular cross-talk between the embryo and the decidua. One of the key molecules during this process is human chorionic gonadotropin (HCG). HCG effectively modulates several metabolic pathways within the decidua contributing to endometrial receptivity. Herein, a brief overview of the molecular mechanisms regulated by HCG is presented. Furthermore, we summarize the existing evidence regarding the clinical impact on reproductive outcomes after endometrial priming with HCG prior to embryo transfer. Although promising, further evidence is needed to clarify the protocol that would lead to beneficial outcomes.

## 1. Introduction

Implantation is the result of a plethora of mechanisms that happen concurrently in a timely synchronized pattern. As the embryo advances to blastocyst, the decidualized endometrium evolves to a receptive phenotype [1]. The biochemical cross-talking between the embryo and the decidua, established in the maternal fetal interface, happens in synchrony. The pre-implantation embryo secretes factors that can modulate the implantation site [2,3]. On the other hand, the decidua secretes cytokines and growth factors that affect embryonic differentiation and early development [2,3]. Under the appropriate biochemical environment, the embryonic and endometrial components finally interact in order for the trophoblast to invade. One of the molecules of the initial array of molecular messages between the blastocyst and the decidua is human chorionic gonadotropin (HCG).

This mini-review aims to summarize the contribution of human chorionic gonadotropin (HCG) to implantation, and to present an overview of the clinical evidence regarding the potential of HCG administration in the uterine cavity as a method of molecular priming.

## 2. Human Chorionic Gonadotrophin (HCG): The Role in Implantation and Early Fetal Development

HCG is the first known hormonal signal of the conceptus. Its mRNA is transcribed as early as the 8-cell stage [4], while the blastocyst expresses HCG before its implantation [5,6]. HCG is increasingly produced after implantation by the syncytiotrophoblast [7]. Significant levels of HCG can already be measured in the maternal blood 10 days after fertilization. The peak of HCG production by the placenta is reached between the 10th and 11th week of gestation; the production then decreases until the 12th week to remain steady from that point on. HCG mediates rescue of the corpus luteum and ensures the ongoing production of progesterone [8].

Hyperglycosylated HCG (HCG-H), an HCG isotype with the same polypeptide structure as HCG and branched oligo-saccharide chains that contain larger N- and O-linked oligosaccharides, has recently been shown to play a different non-steroidogenic role in early pregnancy, as it does not induce progesterone production by human granulosa cells in vitro [9,10]. HCG-H presents a peak during implantation and early trophoblast invasion, being the most abundant HCG variant at that time [11]. Recently, it has been shown that the pattern of serum HCG-H concentration during the first trimester is in line with the HCG-H immunoreactivity of the placenta [12]. HCG-H has been demonstrated to be produced mainly by the cytotrophoblast, inducing extravillous cytotrophoblast cell proliferation and invasion in vitro [13,14]. This may be achieved by inhibiting TGF-β receptor and, thus, trophoblast apoptosis [13]. Additionally, HCG-H was found to be expressed by anchoring villi within the maternal decidua, the extravillous trophoblast invading the decidua, and the endovascular trophoblast cells [12]. The recently described co-localization of HCG-H in the syncytiotrophoblast is proposed to be the end result of rapid HCG-H expression and its accumulation to the adjacent syncytiotrophoblast [12]. Simultaneously, standard HCG induces differentiation of cytotrophoblast to syncytiotrophoblasts [15]. The co-operation of HCG-H and standard HCG is considered a driving force for placentation.

Apart from these fundamental roles in early pregnancy [16], emerging data supports the hypothesis of an additional role of HCG in embryo implantation, through direct effects on the endometrium. An in vivo HCG-induced modulation of the baboon endometrium was observed during the “window of implantation” [17]. Both epithelium and stroma displayed marked responses to intra-uterine HCG administration; a steroid-dependent epithelial plaque reaction in the luminal epithelium was shown followed by a steroid independent up-regulation of glycodelin secretion by the glandular epithelium [17]. Of note is the positive feedback reported between HCG and glycodelin [18]. Additionally, an induction of the decidualization-associated α-smooth muscle cell actin synthesis in stromal fibroblasts was documented [17]. The role for HCG in promoting decidualization was further supported by a study in human stromal fibroblasts [19]. To investigate the direct effects of HCG on the human endometrium, an intra-uterine microdialysis device was developed to measure paracrine mediators within the uterine cavity in vivo [20]. HCG provoked a significant inhibition of intrauterine insulin-like growth factor binding protein-1 (IGF-BP-1) and macrophage colony stimulating factor (M-CSF), while leukemia inhibitory factor (LIF), vascular endothelial growth factor (VEGF), and matrix metalloproteinase-9 (MMP-9) were significantly stimulated [20,21,22]. To the same direction, under the influence of HCG, tissue inhibitors of metalloproteinases have been reported to be downregulated, further supporting a role for HCG in trophoblast invasion [21,23]. Several molecules – expressed by the endometrium – involved in embryo implantation such as galectin-3, homeobox-A10 (HOXA-10), VEGF, and glycodelin have been demonstrated to be induced by HCG [24,25]. At the same time, HCG seems to protect decidualized endometrial stromal cells from oxidative stress-related apoptosis [26], while it is reported to regulate progesterone receptor expression via the ERK1/2 pathways [27]. Taken together, all of the above support the thesis that HCG demonstrates important paracrine effects on decidualization, implantation, vascularization, and tissue remodeling. However, emerging evidence questions the mode of pro-implantation action of HCG on human endometrium. Aiming to simulate HCG administration during controlled ovarian stimulation, Evans and Salamonsen studied the prolonged low-dose HCG-exposure of endometrial epithelial cells [28]. Using endometrial samples from women that underwent assisted reproduction techniques (ART) cycles vs. fertile women with normal cycles and applying cell culture techniques, it was observed that prolonged low-dose HCG treatment down-regulated LH-HCG-receptor, triggered LH-HCG-receptor internalization, and desensitized LH-CH-receptor down-stream signaling, especially after a subsequent acute high dose of HCG—the latter simulating the blastocyst-derived HCG [28]. As a result, endometrial cell adhesion ability and tight junction regulation were impaired [28]. According to the authors, such findings could elucidate the mechanism of implantation failures in in vitro fertilization (IVF).

In addition, HCG contributes to the maternal tolerance of the embryo through interactions with immune cells within the receptive endometrium at the time of implantation. HCG has been associated with T cell modulation. In a recent study, Schumacher et al. used migration assays to demonstrate that regulatory T cells (Treg) were attracted by HCG-producing trophoblasts [29]. More importantly, HCG was recently reported to be involved in Treg differentiation [30]. In addition, HCG was found to adjust the T helper (Th) 1/T helper (Th) 2 balance, since HCG inhibited the development of Th1 autoimmune diabetes in a mouse model [31]. HCG has also been implicated—among other trophoblast-secreted molecules—in inhibiting T lymphocytes [32]. Moreover, HCG can induce macrophage migration inhibitory factor (MIF) expression by endometrial stromal cells, mainly by regulating MIF transcription [33]. In that view, HCG can regulate macrophage migration in the maternal-fetal interface. Additionally, HCG can promote innate functions of macrophages, such as the resolution of inflammation [34]. HCG also intervenes with the development of local immune tolerance in the maternal-fetal interface via the Fas/Fas ligand (Fas/FasL)-mediated apoptotic pathway. It was shown that HCG increased apoptosis in endometrial cells, this being attributed to an FasL up-regulation [35].

Finally, HCG has been described as a regulator of uNK cell proliferation, mediated via the mannose receptor (CD206) rather than by the classical LH/HCG receptor that was not expressed [36].

## 3. HCG and Clinical Applications in Assisted Reproduction Techniques: Where We Stand

The effects of HCG on human endometrium constitute the theoretical basis to develop clinical research protocols aiming to investigate HCG efficacy in improving clinical parameters of assisted reproduction protocols. In that view, since 2011, several reports have been published upon this issue with conflicting results [37,38,39,40,41,42]. Although randomized controlled trials with proper design have been conducted, the published evidence up to now has featured large heterogeneity. HCG is administered intra-uterine before either blastocyst transfer or earlier embryo transfer. Additionally, HCG used was either isolated by urine or was produced via recombinant technologies. To further increase the heterogeneity, HCG was used in different concentrations and to different time-points prior to embryo transfer. Taking all these into consideration, it is rather difficult to extract a definite conclusion.

The lack of concordance led to meta-analytic efforts, hoping to highlight the overall result of the HCG intra-uterine administration. Due to heterogeneity, even the meta-analyses must be critically considered. The first meta-analysis by Ye et al. included five randomized controlled trials (RCTs) with 1387 participants randomized as a study (HCG) group (*n* = 680) and a control group (*n* = 707) [43]. The results were promising, since patients who received intra-uterine HCG performed significantly better in terms of biochemical, clinical, and ongoing pregnancy rates compared to controls [43]. However, the initial enthusiasm provoked by this meta-analysis did not last, since a large randomized trial, published immediately afterwards with 1186 IVF cycles randomized to HCG intra-uterine administration and controls, did not show any significant difference in the case of blastocyst transfer [41]. More importantly, this finding was independent of blastocyst quality and of HCG administration timing (either two days or immediately prior to embryo transfer). The size of that study was large enough for a second meta-analysis to be performed and published later on by Osman et al. [44]. This included eight studies with 3087 participants, randomized as a study (HCG) group (*n* = 1614) and a control group (*n* = 1473). The conclusion did not support any HCG superiority, since no significant difference was found in terms of live birth rates between the study and control groups.

Both meta-analyses published recently still include studies with different methodological details as far as type of embryo, HCG concentration, and timing are concerned. Such sources of possible bias have been addressed by a recent Cochrane review [45]. The authors considered an overall meta-analysis on live birth and clinical pregnancy rates to be rather impossible due to high heterogeneity. By identifying the type of embryo transferred (blastocyst or cleavage-stage) and the HCG dose (less than 500 or 500 IU and higher) as the sources of heterogeneity, they proceeded to consider a sub-group meta-analysis. The results supported HCG administration only in the case of cleavage stage embryos transferred to patients primed with an HCG dose of 500 IU or higher.

The physiology of such findings was very recently addressed by investigating the endometrial receptivity of oocyte donors after HCG intra-uterine administration compared to controls [46]. The study protocol was oriented to simulate the endometrium in case of blastocyst transfer. In that view, oocyte donors were administered either HCG (500 IU) or embryo culture medium 3 days after oocyte retrieval, and endometrial sampling was performed 2 days later (day 5 after oocyte retrieval). In agreement with the results of the recent meta-analyses, the assessment of the endometrium with the endometrial receptivity array (transcriptome analysis) did not reveal a significant change in the receptivity profile after HCG administration [46]. Interestingly, HCG succeeded in delaying endometrial stroma advancement noted in the case of controlled ovarian stimulation, contributing to endometrial synchronization [46]. However, it should be noted that the whole experimental setting involved donors with normal endometria. Whether such findings still hold in the case of infertile women needs properly designed studies to be elucidated.

## 4. The Clinical Impact of Intrauterine Administration of HCG-Treated Autologous Peripheral Blood Mononuclear Cells on Repeated Implantation Failures

The idea of immunomodulation of the endometrium prior to embryo transfer has been very appealing, especially in cases of repeated implantation failures. With the aim of provoking an immune reaction, several efforts have been made by transferring autologous peripheral blood mononuclear cells (PBMCs) in the uterine cavity prior to embryo transfer. PBMCs have been transferred either unstimulated or stimulated by priming the PBMCs with immunomodulatory agents [47,48]. In this context, HCG was the first agent to be used for PBMCs’ activation [48]. Few reports exist so far with promising results in treating patients with repeated implantation failure; it seems that endometrial priming with HCG-treated PBMCs prior to embryo transfer improves reproductive outcomes, especially in cases of more than three implantation failures [48,49,50].

## 5. Conclusions

The clinical data taken together with the knowledge stemming from in vitro or ex vivo reports imply a role for HCG in implantation and early trophoblast invasion. However, it is anticipated that the impact of HCG on a successful pregnancy is the end result of a complicated orchestration of events. The HCG cross-talk between the embryo and the decidua seems to need continuous HCG presence, a fact not well-simulated by single-dose HCG intrauterine administration protocols [46]. Perhaps this is the reason for the recent failures to demonstrate HCG superiority in the case of blastocyst transfer. The promising results yielded by early cleavage embryos may be attributed to the achievement of ongoing HCG presence, where the initial HCG administration is followed by HCG secretion from the transferred embryo, sustaining the HCG effects that favor implantation. A consensus is therefore needed to organize a properly powered multi-centric randomized trial involving cleavage embryos transferred to high dose HCG-primed endometria. The produced results, if promising, are then to be verified in women with repeated implantation failures. As far as blastocyst transfer is concerned and in the view of the need for a constant HCG effect, it should be tested whether pregnancy outcomes improve by replacing single HCG treatments with a repetitive administration scheme.

## Abbreviations

ART	Assisted reproduction techniques
ERK 1/2	Extracellular signal-regulated protein kinases 1/2
HCG	Human chorionic gonadotropin
HOXA10	Homeobox A10
LH	Luteinizing gormone
LIF	Leukemia inhibitory factor
M-CSF	Macrophage colony stimulating factor
PBMC	Peripheral blood mononuclear cells
Treg	T regulatory cells
uNK	Uterine natural killer cells
VEGF	Vascular endothelial growth factor

## References

[1] Herington J.L., Guo Y., Reese J., Paria B.C. (2016). Gene profiling the window of implantation: Microarray analyses from human and rodent models. J. Reprod. Health Med..

[2] Salamonsen L.A., Evans J., Nguyen H.P., Edgell T.A. (2016). The microenvironment of human implantation: Determinant of reproductive success. Am. J. Reprod. Immunol..

[3] Sharma S., Godbole G., Modi D. (2016). Decidual control of trophoblast invasion. Am. J. Reprod. Immunol..

[4] Jurisicova A., Antenos M., Kapasi K., Meriano J., Casper R.F. (1999). Variability in the expression of trophectodermal markers β -human chorionic gonadotrophin, human leukocyte antigen-G and pregnancy specific β -1 glycoprotein by the human blastocyst. Hum. Reprod..

[5] Bonduelle M.L., Dodd R., Liebaers I., Van Steirteghem A., Williamson R., Akhurst R. (1988). Chorionic gonadotrophin-β mrna, a trophoblast marker, is expressed in human 8-cell embryos derived from tripronucleate zygotes. Hum. Reprod..

[6] Lopata A., Hay D.L. (1989). The potential of early human embryos to form blastocysts, hatch from their zona and secrete hCG in culture. Hum. Reprod..

[7] Hoshina M., Boothby M., Hussa R., Pattillo R., Camel H.M., Boime I. (1985). Linkage of human chorionic gonadotrophin and placental lactogen biosynthesis to trophoblast differentiation and tumorigenesis. Placenta.

[8] Shikone T., Yamoto M., Kokawa K., Yamashita K., Nishimori K., Nakano R. (1996). Apoptosis of human corpora lutea during cyclic luteal regression and early pregnancy. J. Clin. Endocrinol. Metab..

[9] Crochet J.R., Shah A.A., Schomberg D.W., Price T.M. (2012). Hyperglycosylated human chorionic gonadotropin does not increase progesterone production by luteinized granulosa cells. J. Clin. Endocrinol. Metab..

[10] Cole L.A. (2012). hCG, five independent molecules. Clin. Chim. Acta.

[11] Sasaki Y., Ladner D.G., Cole L.A. (2008). Hyperglycosylated human chorionic gonadotropin and the source of pregnancy failures. Fertil. Steril..

[12] Evans J., Salamonsen L.A., Menkhorst E., Dimitriadis E. (2015). Dynamic changes in hyperglycosylated human chorionic gonadotrophin throughout the first trimester of pregnancy and its role in early placentation. Hum. Reprod..

[13] Cole L.A. (2010). Hyperglycosylated hCG, a review. Placenta.

[14] Guibourdenche J., Handschuh K., Tsatsaris V., Gerbaud P., Leguy M.C., Muller F., Brion D.E., Fournier T. (2010). Hyperglycosylated hCG is a marker of early human trophoblast invasion. J. Clin. Endocrinol. Metab..

[15] Shi Q.J., Lei Z.M., Rao C.V., Lin J. (1993). Novel role of human chorionic gonadotropin in differentiation of human cytotrophoblasts. Endocrinology.

[16] Keay S.D., Vatish M., Karteris E., Hillhouse E.W., Randeva H.S. (2004). The role of hCG in reproductive medicine. BJOG.

[17] Fazleabas A.T., Donnelly K.M., Srinivasan S., Fortman J.D., Miller J.B. (1999). Modulation of the baboon (*Papio anubis*) uterine endometrium by chorionic gonadotrophin during the period of uterine receptivity. Proc. Natl. Acad. Sci. USA.

[18] Toth B., Roth K., Kunert-Keil C., Scholz C., Schulze S., Mylonas I., Friese K., Jeschke U. (2008). Glycodelin protein and mrna is downregulated in human first trimester abortion and partially upregulated in mole pregnancy. J. Histochem. Cytochem..

[19] Han S.W., Lei Z.M., Rao C.V. (1999). Treatment of human endometrial stromal cells with chorionic gonadotropin promotes their morphological and functional differentiation into decidua. Mol. Cell. Endocrinol..

[20] Licht P., Losch A., Dittrich R., Neuwinger J., Siebzehnrubl E., Wildt L. (1998). Novel insights into human endometrial paracrinology and embryo-maternal communication by intrauterine microdialysis. Hum. Reprod. Update.

[21] Fluhr H., Bischof-Islami D., Krenzer S., Licht P., Bischof P., Zygmunt M. (2008). Human chorionic gonadotropin stimulates matrix metalloproteinases-2 and -9 in cytotrophoblastic cells and decreases tissue inhibitor of metalloproteinases-1, -2, and -3 in decidualized endometrial stromal cells. Fertil. Steril..

[22] Fluhr H., Carli S., Deperschmidt M., Wallwiener D., Zygmunt M., Licht P. (2008). Differential effects of human chorionic gonadotropin and decidualization on insulin-like growth factors-i and -ii in human endometrial stromal cells. Fertil. Steril..

[23] Tapia-Pizarro A., Argandona F., Palomino W.A., Devoto L. (2013). Human chorionic gonadotropin (hCG) modulation of TIMP1 secretion by human endometrial stromal cells facilitates extravillous trophoblast invasion in vitro. Hum. Reprod..

[24] Fogle R.H., Li A., Paulson R.J. (2010). Modulation of hoxa10 and other markers of endometrial receptivity by age and human chorionic gonadotropin in an endometrial explant model. Fertil. Steril..

[25] Yang H., Lei C.X., Zhang W. (2013). Human chorionic gonadotropin (hCG) regulation of galectin-3 expression in endometrial epithelial cells and endometrial stromal cells. Acta Histochem..

[26] Kajihara T., Uchino S., Suzuki M., Itakura A., Brosens J.J., Ishihara O. (2011). Human chorionic gonadotropin confers resistance to oxidative stress-induced apoptosis in decidualizing human endometrial stromal cells. Fertil. Steril..

[27] Tapia-Pizarro A., Archiles S., Argandona F., Valencia C., Zavaleta K., Cecilia Johnson M., Gonzalez-Ramos R., Devoto L. (2017). hCG activates epac-Erk1/2 signaling regulating progesterone receptor expression and function in human endometrial stromal cells. Mol. Hum. Reprod..

[28] Evans J., Salamonsen L.A. (2013). Too much of a good thing? Experimental evidence suggests prolonged exposure to hCG is detrimental to endometrial receptivity. Hum. Reprod..

[29] Schumacher A., Brachwitz N., Sohr S., Engeland K., Langwisch S., Dolaptchieva M., Alexander T., Taran A., Malfertheiner S.F., Costa S.D. (2009). Human chorionic gonadotropin attracts regulatory t cells into the fetal-maternal interface during early human pregnancy. J. Immunol..

[30] Diao L.H., Li G.G., Zhu Y.C., Tu W.W., Huang C.Y., Lian R.C., Chen X., Li Y.Y., Zhang T., Huang Y. (2017). Human chorionic gonadotropin potentially affects pregnancy outcome in women with recurrent implantation failure by regulating the homing preference of regulatory T cells. Am. J. Reprod. Immunol..

[31] Khan N.A., Khan A., Savelkoul H.F., Benner R. (2001). Inhibition of diabetes in nod mice by human pregnancy factor. Hum. Immunol..

[32] Dong M., Ding G., Zhou J., Wang H., Zhao Y., Huang H. (2008). The effect of trophoblasts on T lymphocytes: Possible regulatory effector molecules--A proteomic analysis. Cell. Physiol. Biochem..

[33] Akoum A., Metz C.N., Morin M. (2005). Marked increase in macrophage migration inhibitory factor synthesis and secretion in human endometrial cells in response to human chorionic gonadotropin hormone. J. Clin. Endocrinol. Metab..

[34] Wan H., Versnel M.A., Cheung W.Y., Leenen P.J., Khan N.A., Benner R., Kiekens R.C. (2007). Chorionic gonadotropin can enhance innate immunity by stimulating macrophage function. J. Leukoc. Biol..

[35] Kayisli U.A., Selam B., Guzeloglu-Kayisli O., Demir R., Arici A. (2003). Human chorionic gonadotropin contributes to maternal immunotolerance and endometrial apoptosis by regulating Fas-Fas ligand system. J. Immunol..

[36] Kane N., Kelly R., Saunders P.T., Critchley H.O. (2009). Proliferation of uterine natural killer cells is induced by human chorionic gonadotropin and mediated via the mannose receptor. Endocrinology.

[37] Dehghani Firouzabadi R., Janati S., Razi M.H. (2016). The effect of intrauterine human chorionic gonadotropin injection before embryo transfer on the implantation and pregnancy rate in infertile patients: A randomized clinical trial. Int. J. Reprod. Biomed..

[38] Mansour R., Tawab N., Kamal O., El-Faissal Y., Serour A., Aboulghar M., Serour G. (2011). Intrauterine injection of human chorionic gonadotropin before embryo transfer significantly improves the implantation and pregnancy rates in in vitro fertilization/intracytoplasmic sperm injection: A prospective randomized study. Fertil. Steril..

[39] Navali N., Gassemzadeh A., Farzadi L., Abdollahi S., Nouri M., Hamdi K., Mallah F., Jalilvand F. (2016). Intrauterine administration of hCG immediately after oocyte retrieval and the outcome of ICSI: A randomized controlled trial. Hum. Reprod..

[40] Santibanez A., Garcia J., Pashkova O., Colin O., Castellanos G., Sanchez A.P., De la Jara J.F. (2014). Effect of intrauterine injection of human chorionic gonadotropin before embryo transfer on clinical pregnancy rates from in vitro fertilisation cycles: A prospective study. Reprod. Biol. Endocrinol..

[41] Wirleitner B., Schuff M., Vanderzwalmen P., Stecher A., Okhowat J., Hradecky L., Kohoutek T., Kralickova M., Spitzer D., Zech N.H. (2015). Intrauterine administration of human chorionic gonadotropin does not improve pregnancy and life birth rates independently of blastocyst quality: A randomised prospective study. Reprod. Biol. Endocrinol..

[42] Zarei A., Parsanezhad M.E., Younesi M., Alborzi S., Zolghadri J., Samsami A., Amooee S., Aramesh S. (2014). Intrauterine administration of recombinant human chorionic gonadotropin before embryo transfer on outcome of in vitro fertilization/ intracytoplasmic sperm injection: A randomized clinical trial. Iran. J. Reprod. Med..

[43] Ye H., Hu J., He W., Zhang Y., Li C. (2015). The efficacy of intrauterine injection of human chorionic gonadotropin before embryo transfer in assisted reproductive cycles: Meta-analysis. J. Int. Med. Res..

[44] Osman A., Pundir J., Elsherbini M., Dave S., El-Toukhy T., Khalaf Y. (2016). The effect of intrauterine hCG injection on IVF outcome: A systematic review and meta-analysis. Reprod. Biomed. Online.

[45] Craciunas L., Tsampras N., Coomarasamy A., Raine-Fenning N. (2016). Intrauterine administration of human chorionic gonadotropin (hCG) for subfertile women undergoing assisted reproduction. Cochrane Database Syst. Rev..

[46] Strug M.R., Su R., Young J.E., Dodds W.G., Shavell V.I., Diaz-Gimeno P., Ruiz-Alonso M., Simon C., Lessey B.A., Leach R.E. (2016). Intrauterine human chorionic gonadotropin infusion in oocyte donors promotes endometrial synchrony and induction of early decidual markers for stromal survival: A randomized clinical trial. Hum. Reprod..

[47] Makrigiannakis A., BenKhalifa M., Vrekoussis T., Mahjub S., Kalantaridou S.N., Gurgan T. (2015). Repeated implantation failure: A new potential treatment option. Eur. J. Clin. Investig..

[48] Yoshioka S., Fujiwara H., Nakayama T., Kosaka K., Mori T., Fujii S. (2006). Intrauterine administration of autologous peripheral blood mononuclear cells promotes implantation rates in patients with repeated failure of IVF-embryo transfer. Hum. Reprod..

[49] Li S., Wang J., Cheng Y., Zhou D., Yin T., Xu W., Yu N., Yang J. (2017). Intrauterine administration of hCG-activated autologous human peripheral blood mononuclear cells (PBMC) promotes live birth rates in frozen/thawed embryo transfer cycles of patients with repeated implantation failure. J. Reprod. Immunol..

[50] Yu N., Zhang B., Xu M., Wang S., Liu R., Wu J., Yang J., Feng L. (2016). Intrauterine administration of autologous peripheral blood mononuclear cells (PBMCS) activated by hCG improves the implantation and pregnancy rates in patients with repeated implantation failure: A prospective randomized study. Am. J. Reprod. Immunol..

